# Functional plurihormonal adrenal oncocytoma: case report and literature review

**DOI:** 10.1002/ccr3.1279

**Published:** 2017-11-22

**Authors:** Pablo René Costanzo, Andrea Laura Paissan, Pablo Knoblovits

**Affiliations:** ^1^ Servicio de Endocrinología, Metabolismo y Medicina Nuclear Hospital Italiano de Buenos Aires Buenos Aires Argentina

**Keywords:** Adrenal gland, adrenal oncocytic carcinoma, adrenal oncocytic neoplasm, adrenal oncocytoma, oncocytoma

## Abstract

We present a 27‐year‐old woman with an adrenal oncocytoma. This is a very rare entity. We provide a review of the clinical, biochemical and pathological features of cases reported in the literature.

## Introduction

Oncocytomas or oncocytic neoplasms are composed of tumor cells that show abundant granular eosinophilic cytoplasm by microscopy, and which may adopt an alveolar, tubular, or solid pattern 1. Ultrastructurally, granular cytoplasm is caused by massive accumulation of mitochondria 2.

The organs most commonly involved are the kidney, salivary glands, and thyroid gland. However, cases have been reported in parathyroid glands, pituitary gland, larynx, liver, breast, ovary, stomach, small intestine, thymus, prostate, and lung 1, 3, 4, 5, 6, 7.

When located in the retroperitoneum, oncocytic neoplasms may be either renal or adrenal in origin. Limited cases of retroperitoneal heterotopic adrenal oncocytomas have been reported 8, 9, 10.

Oncocytic neoplasms arising from the adrenal gland are rare: 1.8% of adrenal masses, predominantly affecting the adult population 11. Most of these tumors involve the adrenocortical region, while oncocytic pheochromocytomas are extremely rare 12.

Since the first description of oncocytomas in 1986, confirmed by electron microscopy 13, approximately 147 cases have been reported in the literature 14, most of them described as adrenal incidentalomas. They have been traditionally considered as benign and nonfunctional tumors, although recent data suggest that 20% of oncocytomas show some elements of malignancy and 10–20% are funcional 15.

According to their biologic behavior, oncocytomas are classified as malignant, borderline (or of uncertain malignant potential), and benign, based on the criteria proposed in 2004 by Bisceglia et al. 15. This classification includes major criteria: venous invasion, presence of atypical mitoses, and a high mitotic rate (more than five mitoses per 50 high‐power fields) and minor criteria: presence of necrosis, size >10 centimeters and weight >200 g, capsular invasion, or sinusoidal invasion. The presence of at least one major criterion indicates malignancy, while the presence of at least one minor criterion indicates uncertain malignant potential (borderline). The absence of major and minor criteria is indicative of benign oncocytoma.

As oncocytoma is a very rare entity, the aim of this article was to report a case of plurihormonal adrenal oncocytoma and to provide a review of the clinical, biochemical, and pathological features of cases reported in the literature.

## Material and Methods

We present a case report and a systematic literature review of articles published over the 29‐year period between 1986 and 2015. OVID MEDLINE and PubMed were used to identify relevant articles. The key words “oncocytoma,” “adrenal gland,” “adrenal oncocytoma,” “adrenal oncocytic neoplasm,” and “adrenal oncocytic carcinoma” were used. No language restrictions were imposed. Original articles, review articles, and editorials were included and reviewed in order to select relevant articles. A total of 115 articles were identified. Only full‐text publications or abstracts containing a description of relevant clinical, biochemical, and pathological features were included. Six articles were excluded because the full text could not be obtained and the abstracts contained insufficient data. Data of 109 articles were included in the review, comprising 183 cases of oncocytoma. Data were collected on age, gender, form of presentation, symptoms, hormonal hyperfunction, size, weight, biologic behavior, and progress.

## Case Report

A twenty‐seven‐year‐old woman with no relevant medical history presented with lower back pain of one‐year duration.

She denied any history of medications, smoking, hypertension, headaches, palpitations, excessive perspiration, muscle weakness, bruising easily, fatigue, hematuria, menstrual disorders, acne, or hirsutism.

Physical examination was unremarkable; specifically, no masses, lymphadenopathy, peripheral edema, hirsutism, acne, alopecia, cushingoid features, striae, or petechiae were noted. Blood pressure: 110/70 mmHg, with no orthostatic hypotension.

**Figure 1:**
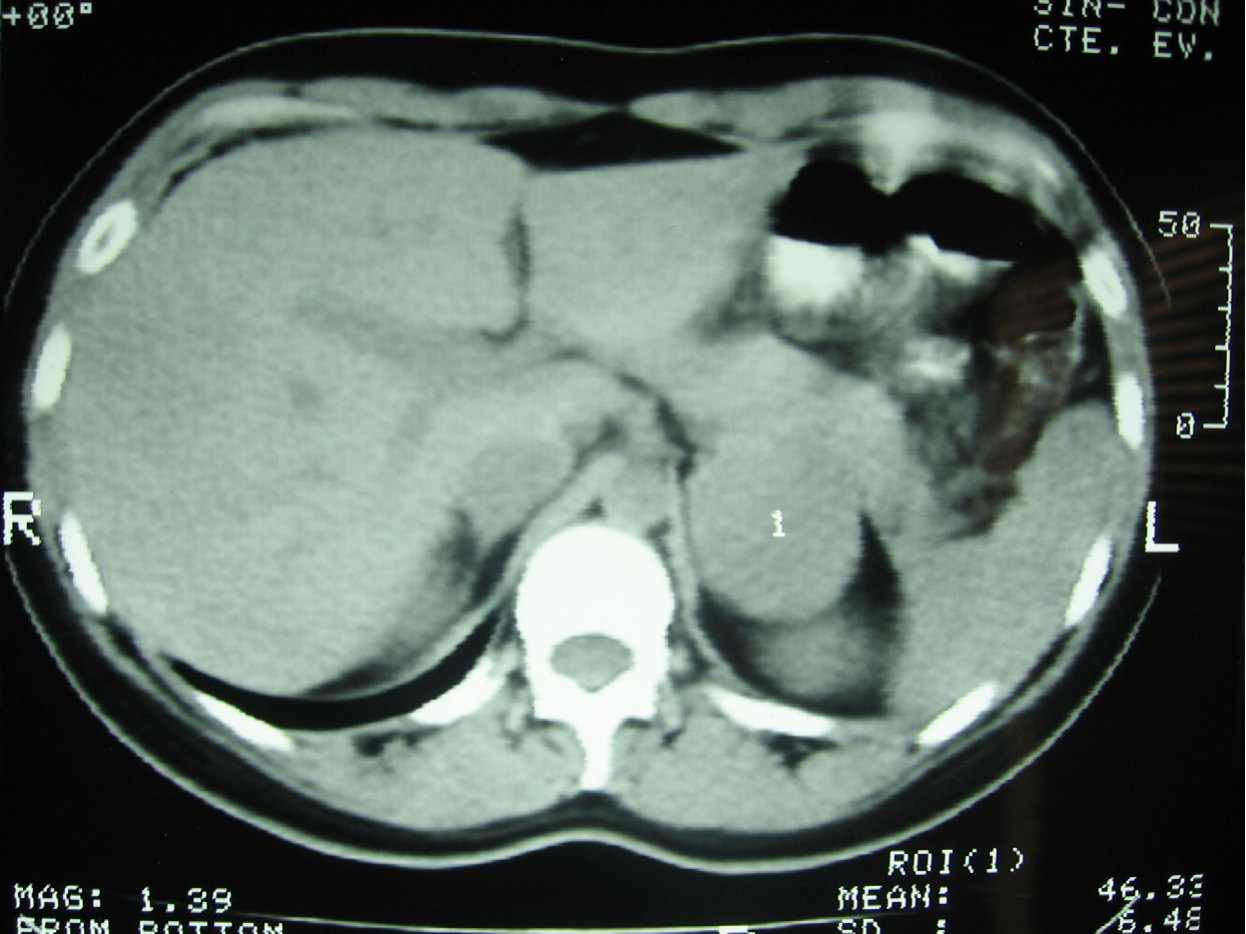
CT scan showed a 50 × 42‐mm rounded, solid, homogeneous expansive lesion of distinct borders in the left adrenal gland.

**Figure 2:**
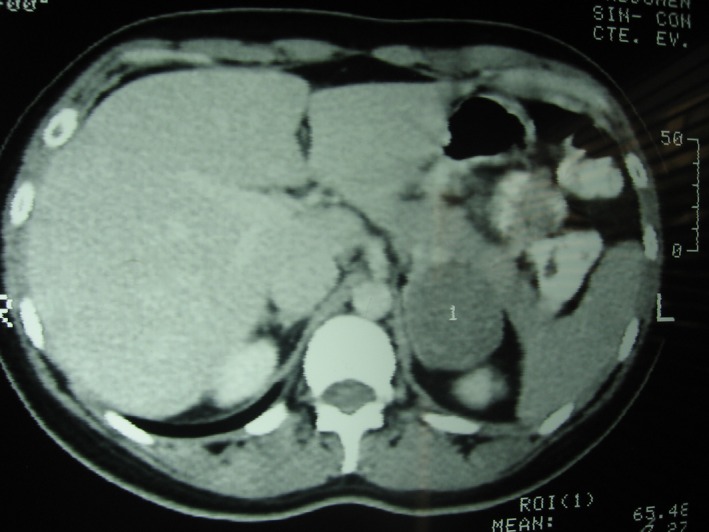
CT scan showed a slightly enhanced tumor in the left adrenal gland.

An ultrasound scan was performed because of the lower back pain and showed a 46 × 40‐mm hypoechoic solid lesion in the left adrenal gland. A CT scan of the abdomen and pelvis was ordered, which demonstrated a 50 × 42‐mm rounded, solid, homogeneous expansive lesion of distinct borders in the left adrenal gland. The lesion had a spontaneous density of 46 Hounsfield units, showing enhancement after contrast administration (Figs 1 and 2).

The patient′s complete blood count, urine analysis, blood urea nitrogen, and creatinine levels were all within normal limits.

Laboratory tests were ordered during the early follicular phase, and at 8 am fasting, the results were as follows: total testosterone: 0.9 ng/mL (normal: 0.1–0.8 ng/mL), DHEA‐S: 2735.3 ng/mL (normal: 201–4142 ng/mL), androstenedione: 3.3 (normal: 0.5–2.7 ng/mL), 17 hydroxyprogesterone: 3.1 ng/mL (normal: 0.3–1.0 ng/mL), aldosterone: 11 ng/dL (normal: 1.2–16 ng/dL, plasma renin activity (PRA): 0.2 ng/mL/h (normal: 0.5–2.6 ng/mL/h), aldosterone/ARP ratio: 55, sodium: 141 mmol/L (normal: 135–145 mmol/L), potassium: 3.9 mmol/L (normal: 3.5–5.3 mmol/L), plasma cortisol: 16.2 *μ*g/dL (normal: 5–25 *μ*g/dL), and ACTH: <10.0 pg/mL (normal: <54 pg/mL). Other measurements were ordered, including salivary cortisol at 23:00 h: 9.3 nmol/L (normal: 0.7–5.0 nmol/L), urinary free cortisol: 73.2 *μ*g/24 h (normal: <100.0 *μ*g/24 h), adrenaline: 4.7 *μ*g/24 h (normal: 0–6.2 *μ*g/24 h), noradrenaline: 14.2 *μ*g/24 h (normal: 11.3–74.3 *μ*g/24 h), and vanillynmandelic acid: 3.8 mg/24 h (normal: <8.5 mg/24 h); 24–h urinary metanephrines measurement was not available in our country at the time of the patient evaluation.

Measurement of nocturnal salivary cortisol was repeated: 5.1 nmol/L (normal: 0.7–5.0 nmol/L). Overnight 1 mg dexamethasone suppression test was ordered: Plasma cortisol levels at 8 am were 16.3 *μ*g/dL (normal <1.8 *μ*g/dL). Endogenous hypercortisolism of adrenal origin was confirmed, associated with biochemical features consistent with elevated aldosterone/PRA ratio (primary aldosteronism not evaluated by confirmatory tests) and hyperandrogenism.

Because of tumor size and plurihormonal secretion, a decision was made to proceed with surgery, which was performed 10 months after diagnosis. A laparoscopic approach was used, but because of the large tumor size, conversion to open surgery was necessary. Left adrenalectomy was performed. No preoperative therapy was performed because there were no clinical or biochemical data suggesting catecholamine hypersecretion; however, careful intraoperative blood pressure monitoring was performed, which remained stable throughout the procedure. The patient made good progress, receiving postoperative hydrocortisone replacement 20 mg/day for 2 months.

### Pathology findings

Left adrenal gland that weighed 134 g and measured 9.9 × 7.5 × 4.9 cm, with a brownish surface. On cut section, the parenchyma was replaced by a brownish‐yellowish tumor proliferation, with areas of hemorrhage and hard‐elastic consistency. Histological sections revealed tumor proliferation composed of polygonal cells of large hyperchromatic bizarre nuclei with isolated intranuclear vacuoles and prominent nucleoli; abundant eosinophilic cytoplasm. Cells were arranged in nests separated by thin fibrous septae; the tumor mass was surrounded by a thick capsule. The mitotic index was low: 2–3 mitoses per 50 high‐power fields. There was no evidence of vascular embolism or capsular invasion. Little residual adrenal parenchyma was observed at the periphery of the lesion. Ki‐67: 3%.

Two months after surgery, hydrocortisone therapy was discontinued and hormonal laboratory measurements were performed, with normal results (Table 1).

**Table 1:** Preoperative and postoperative hormonal laboratory measurements

	Preoperative	Postoperative
Total testosterone (RR: 0.1–0.8 ng/mL)	0.9	<0.1
DHEA‐S (RR: 201–4142 ng/mL)	2735.3	1075.0
Androstenedione (RR: 0.5–2.7 ng/mL)	3.3	1.2
17 hydroxyprogesterone (RR: 0.3–1.0 ng/mL)	3.1	0.5
Aldosterone (RR: 1.2–16 ng/dL)	11	14
PRA (RR: 0.5–2.6 ng/ml/h)	0.2	0.44
Aldosterone/PRA ratio	55	31.8
Plasma cortisol (RR: 5–25 *μ*g%)	16.2	14.3
ACTH (RR: <54 pg/mL)	<10	29
Urinary free cortisol (RR: <100 *μ*g/24 h)	73.2	18.0
Salivary cortisol at 23:00 h (RR: 0.7–5.0 nmol/L)	9.3	1.8

RR, reference range; DHEA‐S, dehydroepiandrosterone sulfate; PRA, plasma renin activity; ACTH, adrenocorticotropic hormone. Blood samples were taken at 8:00 am after 12‐h nocturnal fasting. Aldosterone/PRA ratio was elevated, and not confirmatory test was performed.

The patient made good progress, with no evidence of tumor recurrence on the follow‐up computed tomography scans and laboratory tests performed annually during the last 9 years.

## Discussion

Among adrenal tumors, oncocytomas are a rare subtype. Most cases reported in the literature are isolated cases, with the largest series being that reported by Mearini et al., with a total of 147 cases 14.

We have found 183 case reports of adrenal oncocytomas, from which we could collect clinical, biochemical, and histological data (data are presented as the mean ± SD). These tumors are more frequent in females (2/3) and may occur in all age groups, with a predominant occurrence approximately in the fourth and fifth decades of life (45.1 ± 15.9 years). In agreement with literature reports, about 20% were malignant (22.4%) and about 30% were functional (27.9%). As regards location, previous reviews report a higher frequency on the left side; in this review, 56.2% of tumors occurred on the left side, 38.9% on the right side, 0.6% were bilateral, and 4.3% were heterotopic: retroperitoneum 8, 9, 10, lumbar spine 17, and broad ligament 18. Macroscopically, most of them are large, rounded, encapsulated, and well‐circumscribed tumors. On cut section, they are yellow brownish; some may exhibit areas of hemorrhage or necrosis. Microscopically, they are composed of large eosinophilic and granular cytoplasm cells with a central pyknotic nucleus, with a solid, trabecular, tubular, or papillary growth pattern. The immunohistochemical pattern was difficult to establish because it had not been described in many of the reported cases; these tumors are generally positive for vimentin, melan‐A, synaptophysin, and alpha‐inhibin and negative for chromogranin.

There is consensus that the most important problem regarding adrenal oncocytomas concerns their biologic behavior 15. Aside from obviously malignant cases (unresectable tumors with local invasion or distant metastases), the biologic behavior and prognosis of adrenal tumors are difficult to predict. The tool used for adrenocortical tumors, Weiss score, where nine histologic features are evaluated 19, is not useful because the features that are distinctive of oncocytic tumors (<25% clear cells, nuclear atypia, and diffuse architecture) encompass three criteria of the total; therefore, all oncocytomas would automatically reach the score to be classified as malignant, which is inconsistent with the benign progression observed in the long‐term follow‐up of many reported cases. For this reason, in 2004, Bisceglia et at proposed new criteria, based on a modified Weiss score, for the classification of oncocytic adrenal tumors, suggesting major criteria: venous invasion, presence of atypical mitoses, and a high mitotic rate (more than five mitoses per 50 high‐power fields) and minor criteria: presence of necrosis, size >10 centimeters and weight >200 g, capsular invasion, or sinusoidal invasion. The presence of at least one major criterion indicates malignancy, while the presence of at least one minor criterion indicates uncertain malignant potential (borderline). The absence of major and minor criteria is indicative of benign oncocytoma 15. It is also important to consider the tumor‐proliferating fraction evaluated by immunohistochemistry for Ki 67. Various authors agree that a value >5% characterizes malignancy, while for benign and borderline oncocytomas, a value range between 0 and 4% is reported 15, 20.

Table 2 describes the characteristics of the oncocytomas reported in the literature and the gender and age of patients. Tumors are classified according to their biologic behavior using the criteria proposed by Bisceglia et al. 15.

**Table 2:** Characteristics of oncocytomas according to their biologic behavior

	Benign	Uncertain or Borderline	Malignant
Number	70 (38.3%)	72 (39.3%)	41 (22.4%)
Age (years)	43.1 ± 16.7	44.9 ± 15.4	48.7 ± 15.1
Gender M/F	16/54 (23%/77%)	29/44 (40%/60%)	19/22 (46%/54%)
Functional	26 (37.1%)	11 (15.1%)	14 (34.1%)
Size (cm)	5.1 ± 2.1	11.3 ± 4.7	13.0 ± 6.1
Weight (g)	61.4 ± 53.1	600.0 ± 556.6	940.0 ± 1544.9

Benign oncocytomas constituted 38.3% of all reported cases and were characterized by an average size of 5 cm. In most cases, these tumors were detected incidentally, while in 18.5% of cases patients presented with abdominal pain 12, 21, 22, 23, 24, 25. Other less frequent forms of presentation were hirsutism 12, 26, 27, 28, virilization 29, pseudoprecocious puberty 30, Cushing's syndrome 27, 31, 32, blood hypertension 33, 34, 35, fever 36, pain in lower limbs 16, 17, fatigue 37, 38, and feminization 39.

Oncocytomas of uncertain malignant potential (borderline) accounted for 39.3% of cases, with a larger size on average (11.6 cm). Most cases were incidental findings, while the rest presented with abdominal or lumbar pain (29.2%), virilization (6%) 40, 41, 42, Cushing's syndrome 43, blood hypertension 44, 45, weight loss, hematuria 46, 47, fever, or palpable mass in a lower proportion 48, 49.

As regards malignant oncocytomas (22.4%), the average size was 13 cm. In 21.9% of cases, diagnosis was performed by abdominal pain or palpable mass and in 21.9% of cases by excessive hormone secretion symptoms (36.8%). In 10 cases, there was evidence of metastasis 50, 51, 52, 53, 54, 55, 56, and in two cases, there was invasion into the inferior vena cava 57, 58, while seven cases presented tumor recurrence after resection 55, 56, 59, 60.

As regards imaging methods, in addition to tumor size, the appearance and behavior after contrast administration are important data for the differential diagnosis of adrenal masses. Lipid content, evaluated by CT or magnetic resonance imaging, is the most important parameter for differentiating adrenal adenomas from lesions with malignant behavior, such as adrenal carcinoma or metastases. The majority of adrenal adenomas present attenuation values of less than 10 HU on unenhanced CT, reflecting the abundant intracellular lipid content, while 30% present higher densities and are considered lipid‐poor adenomas. The behavior of lesions after contrast administration allows differentiation of adenomatous lesions from malignant tumors, as the former show a rapid washout of contrast (greater than 50%) on delayed scans, while carcinomas or metastases enhance intensely and retain contrast for longer periods. Khan et al. described a series of benign and malignant adrenal oncocytomas, comparing them with various adrenal carcinomas as to size, values of attenuation on CT scan and behavior of contrast material. Benign oncocytomas were more frequently homogeneous, with no evidence of calcification or hemorrhage. The value of attenuation was greater than 10 HU, which is consistent with the histological feature of poor intracellular lipid content. However, these tumors demonstrated high‐contrast washout percentages, behaving similarly to lipid‐poor adenomas. Oncocytic carcinomas showed similar features to those found in adrenocortical carcinomas, including larger size, heterogeneity, and presence of necrosis. They showed high values of attenuation on unenhanced CT, 39 HU on average, together with a washout percentage <50% after contrast administration 61.

Independently of the size and features of adrenal masses, in all cases it is necessary to perform a thorough hormonal assessment to determine whether there are signs of adrenal hyperfunction. Recommended tests include measurement of plasma or urinary fractionated metanephrines, low‐dose dexamethasone suppression test, measurement of ACTH, estradiol, androgens (DHEA‐S, delta 4 androstenedione, 17 hydroxyprogesterone, and testosterone) aldosterone and plasma renin activity. In our review of the literature, approximately one‐third of benign oncocytomas were functional, secreting androgen in six cases 28, 29, 37, 62, 63, androgens and cortisol in five cases 26, 27, 31, 32, cortisol in six cases 34, 63, 64, estradiol in two cases 30, 39, IL‐6 in two cases 36, 38, aldosterone in four cases 34, 35, 55, and catecholamines in the remaining case 65. Among borderline tumors or tumors of uncertain malignant potential, hormonal hypersecretion was found in 17% of cases: four androgen‐secreting tumors 4, 41, 66, two corticosteroid‐secreting tumors 43, 67, two catecholamine‐secreting tumors 45, 68, one corticosteroid‐ and androgen‐secreting tumor 69, one aldosterone‐secreting tumor 70, and one 17‐ketosteroids‐secreting tumor 71. Among malignant oncocytomas, 33.3% were functional, secreting androgen in four cases 55, 72, cortisol in seven cases 34, 55, 59, androgens and cortisol in one case 54, aldosterone and cortisol in one case 60, and estrogens in one case 55. There were no data available on the progress of patients with cortisol‐secreting tumors, regarding the presence of signs or symptoms of adrenal suppression after surgery.

As adrenal oncocytomas usually present as masses larger than 5 cm, adrenalectomy is the treatment of choice, generally by the open surgical approach, although recently the use of the laparoscopic technique has been increasingly reported in cases of encapsulated tumors. The laparoscopic approach reduces pain and the perioperative hospital stay; however, the indication of this technique in cases of larger or potentially malignant tumors remains controversial 14. After resection, follow‐up will depend on the histological features of the tumor, depending on whether it is benign, of uncertain malignant potential or malignant. There are no general recommendations, but imaging and laboratory follow‐up is suggested for a minimum of 5 years for functional tumors 14.

Our patient presented with lumbar pain as the only clinical manifestation; therefore, it was not an incidentaloma. Although the laboratory findings demonstrated a functional tumor, there were no clinical signs of adrenal hyperfunction. The hormonal evaluation showed ACTH‐independent cortisol secretion and androgen secretion, which returned to normal levels in postoperative laboratory tests. Our patient received short‐term hydrocortisone replacement therapy, with no clinical manifestations of adrenal suppression secondary to resection of the cortisol‐secreting tumor. In most studies that evaluated patients with hyperaldosteronism, an aldosterone/PRA ratio >20 is considered highly suspicious for primary aldosteronism. An aldosterone/PRA ratio >30, especially in the setting of plasma aldosterone concentration ≥15 ng/dL, has been shown to be 90% sensitive and 91% specific for the diagnosis 73, 74. Although the aldosterone/PRA ratio was high in our patient, no confirmatory tests were performed to establish the diagnosis of primary aldosteronism. There is a discrepancy between the size of the resected tumor and that reported in preoperative imaging assessments (by ultrasound and tomography). This might be possibly explained by a rapid tumor growth in the months between diagnosis and resection. However, histopathologically, none of the major or minor criteria proposed by Bisceglia 15 were observed; therefore, contrary to any expectations based on tumor growth rate, our case was classified as a benign oncocytoma, which is consistent with the good progress shown by the patient in the long‐term follow‐up.

In conclusion, adrenal oncocytic tumors are rare entities with a variable biologic behavior, which require a thorough hormonal evaluation for ruling out hyperfunction before surgical resection and for an adequate management in cases of cortisol‐secreting tumors that may cause postoperative contralateral adrenal suppression. The pathologic examination must be performed by experienced pathologists in order to be able to classify tumors according to their biologic potential and to establish the patient's prognosis.

## Authorship

PRC: contributed to patient evaluation, design and conduction of research, data acquisition and analysis, and drafting of the manuscript. ALP: helped in design and conduction of research, data acquisition and analysis, and drafting of the manuscript. PK: contributed to analysis of the data, drafting, and critical review of the manuscript.

## Conflict of Interest

None declared.

## References

[1] Chang, A. , and S. J. Harawi . 1992 Oncocytes, oncocytosis and oncocytic tumors. Pathol. Annu. 27:263–304. 1736246

[2] Ghadially, F. N. 1985 Diagnostic electron microscopy of tumours, 2nd ed Butterworths, London.

[3] Millard, P. R. , and H. M. Bishop . 1984 Oncocytoma of the stomach: a case report. Histopathology 8:1053–1058. 6526387 10.1111/j.1365-2559.1984.tb02420.x

[4] Meijer, S. , and H. F. Hoitsma . 1982 Malignant intrathoracic oncocytoma. Cancer 49:97–100. 7053823 10.1002/1097-0142(19820101)49:1<97::aid-cncr2820490120>3.0.co;2-d

[5] Beer, M. , F. Occhionero , and U. Welsch . 1990 Oncocytoma of the prostate: a case report with ultrastructural and immunohistochemical evaluation. Histopathology 17:370–372. 2175296 10.1111/j.1365-2559.1990.tb00744.x

[6] Brandwein, M. S. , and A. G. Huvos . 1991 Oncocytic tumors of major salivary glands. A study of 68 cases with follow‐up of 44 patients. Am. J. Surg. Pathol. 15:514–528. 2031528 10.1097/00000478-199106000-00002

[7] Amin, M. B. , T. B. Crotty , S. K. Tickoo , G. M. Farrow . 1997 Renal oncocytoma: a reappraisal of morphologic features with clinicopathologic findings in 80 cases. Am. J. Surg. Pathol. 21:1–12. 8990136 10.1097/00000478-199701000-00001

[8] Corsi, A. , M. Riminucci , V. Petrozza , M. T. Collins , M. E. Natale , A. Cancrini , et al. 2002 Incidentally detected giant oncocytoma arising in retroperitoneal heterotopic adrenal tissue. Arch. Pathol. Lab. Med. 126:1118–1122. 12204066 10.5858/2002-126-1118-IDGOAI

[9] Nguyen, G. K. , R. Vriend , D. Ronaghan , W. H. Lakey . 1992 Heterotopic adrenocortical oncocytoma. A case report with light and electron microscopic studies. Cancer 70:2681–2684. 1423199 10.1002/1097-0142(19921201)70:11<2681::aid-cncr2820701119>3.0.co;2-h

[10] Surrey, L. F. , A. A. Thaker , P. J. Zhang , G. Karakousis , and M. D. Feldman . 2012 Ectopic functioning adrenocortical oncocytic adenoma (oncocytoma) with myelolipoma causing virilization. Case Rep. Pathol. 2012:326418. 23094172 10.1155/2012/326418 PMC3474228

[11] Pędziwiatr, M. , M. Wierdak , M. Ostachowski , M. Natkaniec , M. Białas , A. Hubalewska‐Dydejczyk , et al. 2015 Single center outcomes of laparoscopic transperitoneal lateral adrenalectomy—Lessons learned after 500 cases: a retrospective cohort study. Int. J. Surg. 20:88–94. 26074291 10.1016/j.ijsu.2015.06.020

[12] Li, M. , and B. M. Wenig . 2000 Adrenal oncocytic pheochromocytoma. Am. J. Surg. Pathol. 24:1552–1557. 11075859 10.1097/00000478-200011000-00013

[13] Smirnova, E. A. , and I. G. Mikhailov . 1986 Electron microscopic characteristics of oncocytoma of the lung, small intestine and adrenal gland. Arkh. Patol. 48:79–81. 3019283

[14] Mearini, L. , R. Del Sordo , E. Costantini , E. Nunzi , and M. Porena . 2013 Adrenal oncocytic neoplasm: a systematic review. Urol. Int. 91:125–133. 23147196 10.1159/000345141

[15] Bisceglia, M. , O. Ludovico , A. Di Mattia , D. Ben‐Dor , J. Sandbank , G. Pasquinelli , et al. 2004 Adrenocortical oncocytic tumors: report of 10 cases and review of the literature. Int. J. Surg. Pathol. 12:231–243. 15306935 10.1177/106689690401200304

[16] Schittenhelm, J. , F. H. Ebner , P. Harter , A. Bornemann . 2009 Symptomatic intraspinal oncocytic adrenocortical adenoma. Endocr. Pathol. 20:73–77. 19039533 10.1007/s12022-008-9051-1

[17] Cassarino, D. S. , M. Santi , A. Arruda , R. Patrocinio , M. Tsokos , N. Ghatak , et al. 2004 Spinal adrenal cortical adenoma with oncocytic features: report of the first intramedullary case and review of the literature. Int. J. Surg. Pathol. 12:259–264. 15306940 10.1177/106689690401200309

[18] Kasajima, A. , Y. Nakamura , Y. Adachi , Y. Takahashi , F. Fujishima , Y. Chiba , et al. 2014 Oncocytic adrenocortical neoplasm arising from adrenal rest in the broad ligament of the uterus. Pathol. Int. 64:183–188. 24750189 10.1111/pin.12154

[19] Weiss, L. M. 1984 Comparative histologic study of 43 metastasizing and nonmetastasizing adrenocortical tumors. Am. J. Surg. Pathol. 8:163–169. 6703192 10.1097/00000478-198403000-00001

[20] Aubert, S. , A. Wacrenier , X. Leroy , P. Devos , B. Carnaille , C. Proye , et al. 2002 Weiss system revisited. A clinicopathologic and immunohistochemical study of 49 adrenocortical tumors. Am. J. Surg. Pathol. 26:1612–1619. 12459628 10.1097/00000478-200212000-00009

[21] Gandras, E. J. , L. H. Schwartz , D. M. Panicek , G. Levi . 1996 Case report. Adrenocortical oncocytoma: CT and MRI findings. J. Comput. Assist. Tomogr. 20:407–409. 8626901 10.1097/00004728-199605000-00016

[22] Kakimoto, S. , Y. Yushita , T. Sanefuji , A. Kondo , N. Fujishima , M. Kishikawa , et al. 1986 Non‐hormonal adrenocortical adenoma with oncocytoma‐like appearances. Hinyokika Kiyo. 32:757–763. 3751804

[23] Lin, B. T. , S. M. Bonsib , G. W. Mierau , L. M. Weiss , and L. J. Medeiros . 1998 Oncocytic adrenocortical neoplasms: a report of seven cases and review of the literature. Am. J. Surg. Pathol. 22:603–614. 9591731 10.1097/00000478-199805000-00012

[24] Muir, T. E. , J. A. Ferreiro , and J. A. Carney . 1996 Oncocytoma of the adrenal gland. Mod. Pathol. 9:50A. Abstract.

[25] Botsios, D. , K. Blouhos , K. Vasiliadis , A. Asimaki , K. Tsalis , and D. Betsis . 2007 Adrenocortical oncocytoma – a rare tumor of undefined malignant potential: report of a case. Surg. Today 37:612–617. 17593485 10.1007/s00595-006-3458-4

[26] Sahin, S. B. , A. F. Yucel , R. Bedir , S. Ogullar , T. Ayaz , and E. Algun . 2014 Testosterone and cortisol secreting adrenocortical oncocytoma: an unusual case of hirsutism. Case Rep. Endocrinol. 2014:206890. 24716005 10.1155/2014/206890 PMC3970460

[27] Pereira, B. D. , E. S. Rios , R. A. Cabrera , J. Portugal , and L. Raimundo . 2014 Adrenocortical oncocytoma presenting as Cushing`s syndrome: an additional report of a paediatric case. Endocr. Pathol. 25:397–403. 25077961 10.1007/s12022-014-9325-8

[28] Tetsi Nomigni, M. , S. Ouzounian , A. Benoit , J. Vadrot , F. Tissier , S. Renouf , et al. 2015 Steroidogenic enzyme profile in an androgen‐secreting adrenocortical oncocytoma associated with hirsutism. Endocr. Connect. 4:117–127. 26034121 10.1530/EC-15-0014 PMC4453718

[29] Yordanova, G. , V. Iotova , K. Kalchev , K. Ivanov , B. Balev , N. Kolev , et al. 2015 Virilizing adrenal oncocytoma in a 9 year old girl: rare neoplasm with an intriguing postoperative course. J. Pediatr. Endocrinol. Metab. 28:685–690. 25514324 10.1515/jpem-2014-0308

[30] Tahar, G. T. , K. N. Nejib , S. S. Sadok , and L. M. Rachid . 2008 Adrenocortical oncocytoma: a case report and review of literature. J. Pediatr. Surg. 43:E1–E3. 10.1016/j.jpedsurg.2007.12.067 18485928

[31] Geramizadeh, B. , B. Norouzzadeh , S. Bolandparvaz , and S. Sefidbakht . 2008 Functioning adrenocortical oncocytoma: a case report and review of literature. Indian J. Pathol. Microbiol. 51:237–239. 18603692 10.4103/0377-4929.41667

[32] Lee, Y. S. , W. R. Lin , C. K. Chen , Y. W. Pai , and M. Chen . 2011 Functional adrenal oncocytoma presenting as Cushing`s syndrome: case report and literature review. Urol. Sci. 22:166–168.

[33] Lee, Y. S. , W. R. Lin , C. K. Chen , Y. W. Pai , and M. Chen . 2016 Adrenal oncocytoma of uncertain malignant potential: a rare etiology of adrenal incidentaloma. Clin. Case Rep. 4:303–304. 27014458 10.1002/ccr3.486 PMC4771856

[34] Duregon, E. , M. Volante , S. Cappia , A. Cuccurullo , M. Bisceglia , D. D. Wong , et al. 2011 Oncocytic adrenocortical tumors: diagnostic algorithm and mitochondrial DNA profile in 27 cases. Am. J. Surg. Pathol. 35:1882–1893. 21989346 10.1097/PAS.0b013e31822da401

[35] Mete, O. , and S. L. Asa . 2009 Aldosterone‐producing adrenal cortical adenoma with oncocytic change and cytoplasmic eosinophilic globular inclusions. Endocr. Pathol. 20:182–185. 19462261 10.1007/s12022-009-9082-2

[36] Kawahara, Y. , A. Morimoto , A. Onoue , Y. Kashii , N. Fukushima , and Y. Gunji . 2014 Persistent fever and weight loss due to an interleukin‐6 producing adrenocortical oncocytoma in a girl—review of the literature. Eur. J. Pediatr. 173:1107–1110. 24610396 10.1007/s00431-014-2292-8

[37] Gumy‐Pause, F. , M. Bongiovanni , B. Wildhaber , J. J. Jenkins , C. Chardot , and H. Ozsahin . 2008 Adrenocortical oncocytoma in a child. Pediatr. Blood Cancer 50:718–721. 17091483 10.1002/pbc.21090

[38] Akatsu, T. , K. Kameyama , K. Araki , T. Ashizawa , G. Wakabayashi , and M. Kitajima . 2008 Functioning adrenocortical oncocytoma; the first documented case producing interleukin‐6 and review of the literature. J. Endocrinol. Invest. 31:68–73. 18296908 10.1007/BF03345569

[39] Icard, P. , A. Louvel , M. Le Charpentier , and Y. Chapuis . 2001 Adrenocortical tumors with oncocytic cells: benign or malignant? Ann. Chir. 126:249–253. 11340712 10.1016/s0003-3944(01)00497-7

[40] el‐Naggar, A. K. , D. B. Evans , and B. Mackay . 1991 Oncocytic adrenal cortical carcinoma. Ultrastruc. Pathol. 15:549–556. 10.3109/01913129109016262 1755111

[41] Subbiah, S. , U. Nahar , R. Samujh , and A. Bhansali . 2013 Heterosexual precocity: rare manifestation of virilizing adrenocortical oncocytoma. Ann. Saudi Med. 33:294–297. 23793435 10.5144/0256-4947.2013.294 PMC6078526

[42] Lim, Y. J. , S. M. Lee , J. H. Shin , H. C. Koh , and Y. H. Lee . 2010 Virilizing adrenocortical oncocytoma in a child: a case report. J. Korean Med. Sci. 25:1077–1079. 20592902 10.3346/jkms.2010.25.7.1077 PMC2890887

[43] Kabayegit, O. Y. , D. Soysal , G. Oruk , B. Ustaoglu , U. Kosan , S. Solmaz , et al. 2008 Adrenocortical oncocytic neoplasm presenting with Cushing`s syndrome: a case report. J. Med. Case Rep. 2:228. 18620603 10.1186/1752-1947-2-228 PMC2481265

[44] Kitching, P. A. , V. Patel , and R. Harach . 1999 Adrenocortical oncocytoma. J. Clin. Pathol. 52:151–153. 10396247 10.1136/jcp.52.2.151 PMC501064

[45] Sharma, N. , P. N. Dogra , and S. Mathur . 2008 Functional adrenal oncocytoma: a rare neoplasm. Indian J. Pathol. Microbiol. 51:531–533. 19008586 10.4103/0377-4929.43751

[46] Sasano, H. , T. Suzuki , T. Sano , T. Kameya , N. Sasano , and H. Nagura . 1991 Adrenocortical oncocytoma. A true nonfunctioning adrenocortical tumor. Am. J. Surg. Pathol. 15:949–956. 1928551 10.1097/00000478-199110000-00005

[47] Waters, P. R. , G. D. Haselhuhn , W. T. Gunning 3rd , E. R. Phillips , and S. H. Selman . 1997 Adrenocortical oncocytoma: two case reports and review of literature. Urology 49:624–628. 9111640 10.1016/s0090-4295(96)00543-2

[48] Song, S. Y. , S. Park , S. R. Kim , and Y. L. Suh . 2004 Oncocytic adrenocortical carcinomas: a pathological and immunohistochemical study of four cases in comparison with conventional adrenocortical carcinomas. Pathol. Int. 54:603–610. 15260851 10.1111/j.1440-1827.2004.01669.x

[49] Fernandes, H. , and M. Bukelo . 2012 Fine needle aspiration cytology of adrenal oncocytic neoplasm of uncertain malignant potential. J. Clin. Diagn. Res. 6:1448–1449. 23205372 10.7860/JCDR/2012/4256.2384 PMC3471497

[50] Hoang, M. P. , A. G. Ayala , and J. Albores‐Saavedra . 2002 Oncocytic adrenocortical carcinoma: a morphologic, immunohistochemical and ultrastructural study of four cases. Mod. Pathol. 15:973–978. 12218215 10.1038/modpathol.3880638

[51] Tanaka, K. , Y. Kumano , N. Kanomata , M. Takeda , I. Hara , M. Fujisawa , et al. 2004 Oncocytic adrenocortical carcinoma. Urology 64:376–377. 10.1016/j.urology.2004.04.023 15302503

[52] Kurek, R. , R. Von Knobloch , U. Feek , A. Heidenreich , and R. Hofmann . 2001 Local recurrence of an oncocytic adrenocortical carcinoma with ovary metastases. J. Urol. 166:985. 11490264

[53] Juliano, J. J. , R. L. Cody , and J. H. Suh . 2008 Metastatic adrenocortical oncocytoma: a case report. Urol. Oncol. 26:198–201. 18312941 10.1016/j.urolonc.2007.02.008

[54] Son, S. H. , S. W. Lee , B. I. Song , Y. J. Jang , J. Y. Park , S. Y. Jeong , et al. 2014 Recurrence of a functional adrenocortical oncocytoma of borderline malignant potential showing high FDG uptake on 18F‐FDG PET/CT. Ann. Nucl. Med. 28:69–73. 23990396 10.1007/s12149-013-0764-y

[55] Wong, D. D. , D. V. Spagnolo , M. Bisceglia , M. Havlat , D. McCallum , and M. A. Platten . 2011 Oncocytic adrenocortical neoplasms – a clinicopathologic study of 13 new cases emphasizing the importance of their recognition. Hum. Pathol. 42:489–499. 21237489 10.1016/j.humpath.2010.08.010

[56] Argyriou, P. , C. Zisis , N. Alevizopoulos , E. M. Kefaloyannis , C. Gennatas , and C. D. Petraki . 2008 Adrenocortical oncocytic carcinoma with recurrent metastases: a case report and a review of the literature. World J. Surg. Oncol. 6:134. 19091123 10.1186/1477-7819-6-134 PMC2630932

[57] Krishnamurthy, S. , N. G. Ordóñez , T. O. Shelton , A. G. Ayala , and N. Sneige . 2000 Fine‐needle aspiration cytology of a case of oncocytic adrenocortical carcinoma. Diagn. Cytopathol. 22:299–303. 10790237 10.1002/(sici)1097-0339(200005)22:5<299::aid-dc8>3.0.co;2-5

[58] Segal, S. , S. Cytron , S. Shenhav , and O. Gemer . 2001 Adrenocortical oncocytoma in pregnancy. Obstet. Gynecol. 98:916–918. 11704201 10.1016/s0029-7844(01)01320-5

[59] Gołkowski, F. , M. Buziak‐Bereza , B. Huszno , A. Bałdys‐Waligórska , A. Stefańska , A. Budzyński , et al. 2007 The unique case of adrenocortical malignant and functioning oncocytic tumour. Exp. Clin. Endocrinol. Diabetes 115:401–404. 17701888 10.1055/s-2007-967083

[60] Ali, A. E. , and S. J. Raphael . 2007 Functional oncocytic adrenocortical carcinoma. Endocr. Pathol. 18:187–189. 18058268 10.1007/s12022-007-9000-4

[61] Khan, M. , E. M. Caoili , M. S. Davenport , A. Poznanski , I. R. Francis , T. Giordano , et al. 2014 CT imaging characteristics of oncocytic adrenal neoplasms (OANs): comparison with adrenocortical carcinomas. Abdom. Imaging 39:86–91. 24271078 10.1007/s00261-013-0047-z

[62] Erlandson, R. A. , and V. E. Reuter . 1991 Oncocytic adrenal cortical adenoma. Ultrastruct. Pathol. 15:539–547. 1721751 10.3109/01913129109016261

[63] Ciprová, V. , C. Povýsil , D. Dudorkinová , L. Safarík , and T. Zelinka . 2004 Oncocytic adrenocortical neoplasms. Cesk. Patol. 40:102–105. 15493417

[64] Xiao, G. Q. , D. S. Pertsemlidis , and P. D. Unger . 2005 Functioning adrenocortical oncocytoma: a case report and review of the literature. Ann. Diagn. Pathol. 9:295–297. 16198960 10.1016/j.anndiagpath.2005.05.005

[65] Goel, T. , J. Thomas , S. Garg , A. C. Rao , and S. Reddy . 2007 Adrenal oncocytoma masquerading as a functional tumor. Indian J. Urol. 23:77–78. 19675771 10.4103/0970-1591.30275 PMC2721505

[66] Sharma, D. , S. Sharma , A. Jhobta , and R. G. Sood . 2012 Virilizing adrenal oncocytoma. J. Clin. Imaging Sci. 2:76. 23393632 10.4103/2156-7514.104309 PMC3551489

[67] Alexander, A. , and K. P. Paulose . 1998 Oncocytic variant of adrenal carcinoma presenting as Cushing`s syndrome. J. Assoc. Physicians India 46:235–237. 11273124

[68] Kiriakopoulos, A. , D. Papaioannou , and D. Linos . 2011 Adrenal cortical oncocytoma mimicking pheochromocytoma. Hormones (Athens) 10:76–79. 21349810 10.14310/horm.2002.1296

[69] Logasundaram, R. , C. Parkinson , P. Donaldson , and P. E. Coode . 2007 Co‐secretion of testosterone and cortisol by a functional adrenocortical oncocytoma. Histopathology 51:418–420. 17727488 10.1111/j.1365-2559.2007.02780.x

[70] Terui, K. , S. Sakihara , K. Kageyama , T. Nigawara , S. Takayasu , Y. Matsuhashi , et al. 2010 A case of adrenocortical oncocytoma occurring with aldosteronoma. J. Clin. Endocrinol. Metab. 95:3597–3598. 20685888 10.1210/jc.2009-2787

[71] Lee, R. , H. A. Al‐Ahmadie , S. A. Boorjian , R. R. Gonzalez , C. Badillo , F. Badillo , et al. 2006 A case of incidental adrenocortical oncocytoma. Nat. Clin. Pract. Urol. 3:618–621. 17088930 10.1038/ncpuro0631

[72] Mwandila, M. , H. Waller , V. Stott , and P. Mercer . 2010 A case of testosterone‐secreting oncocytic adrenocortical carcinoma. N. Z. Med. J. 123:80–82. 21317966

[73] Funder, J. W. , R. M. Carey , F. Mantero , M. H. Murad , M. Reincke , H. Shibata , et al. 2016 The management of primary aldosteronism: case detection, diagnosis, and treatment: an endocrine society clinical practice guideline. J. Clin. Endocrinol. Metab. 101:1889–1916. 26934393 10.1210/jc.2015-4061

[74] Young, W. F. 2007 Primary aldosteronism: renaissance of a syndrome. Clin. Endocrinol. (Oxf) 66:607–618. 17492946 10.1111/j.1365-2265.2007.02775.x

